# *Ilex kudingcha* C.J. Tseng (Kudingcha) has *in vitro* anticancer activities in MCF-7 human breast adenocarcinoma cells and exerts anti-metastatic effects *in vivo*

**DOI:** 10.3892/ol.2013.1253

**Published:** 2013-03-14

**Authors:** XIN ZHAO, QIANG WANG, YU QIAN, JIA-LE SONG

**Affiliations:** 1 Department of Biological and Chemical Engineering, Chongqing University of Education, Chongqing 400067, P.R. China;; 2 Department of Food Science and Nutrition, Pusan National University, Busan 609-735, Republic of Korea

**Keywords:** Kudingcha, anticancer, apoptosis, anti-inflammation

## Abstract

*Ilex kudingcha* C.J. Tseng (Kudingcha) is a traditional Chinese drink consumed in East Asia. The present study evaluated the *in vitro* anticancer effects of Kudingcha in MCF-7 human breast adenocarcinoma cells using a 3-(4,5-dimethyl-2-thiazolyl)-2,5-diphenyltetrazolium bromide (MTT) assay. At a concentration of 200 *μ*g/ml, Kudingcha inhibited the growth of the MCF-7 cells by 81%. This was a greater degree of inhibition than that observed at concentrations of 100 and 50 *μ*g/ml (58 and 19%, respectively). To elucidate the inhibitory mechanisms underlying the anticancer effect of Kudingcha in cancer cells, the expression of genes associated with apoptosis and inflammation were measured using RT-PCR. Kudingcha significantly induced apoptosis, as determined by 4,6-diamidino-2-phenylindole (DAPI) staining, by upregulating Bax, caspase-3 and caspase-9, and downregulating Bcl-2. The expression of the NF-κB, iNOS and COX-2 genes associated with inflammation was significantly decreased (P<0.05) by Kudingcha, thus demonstrating its anti-inflammatory properties. Kudingcha has been reported to exhibit inhibitory effects of tumor metastasis induced in 26-M3.1 colon carcinoma cells in BALB/c mice. The results demonstrated that Kudingcha had potent *in vitro* anticancer effects; it induced apoptosis, had anti-inflammatory activities and exerted *in vivo* anti-metastatic effects. Additionally, the anticancer, anti-inflammatory and anti-metastatic effects of Kudingcha were stronger at high concentrations than at low concentrations.

## Introduction

*Ilex kudingcha* C.J. Tseng (Kudingcha) is a bitter tea of Chinese origin. Kudingcha has been consumed traditionally as a type of herbal tea in China and South Eastern Asia (1). *Ilex kudingcha* is one of the main plants that produces Kudingcha in China. Certain studies have investigated its chemical composition and pharmaceutical functions, which demonstrated the numerous functional compositions of Kudingcha and the functional effects of those compositions, such as the antioxidant effect (2). It has been reported that Kudingcha is rich in polyphenolic compounds and that it demonstrates potent antioxidant activities *in vitro*(3,4). It also has been demonstrated that the major phenolic compounds in Kudingcha are caffeoylquinic acid (CQA) derivatives. CQA derivatives are natural functional compounds isolated from a variety of plants, which possess a broad range of pharmacological properties, including antioxidant, hepatoprotectant, antibacterial, antihistaminic, anticancer, neuroprotective and other biological effects (5,6).

Apoptosis induction in cancer cells is initially identified by morphological changes, including cell shrinkage, membrane blebbing, chromatin condensation and nuclear fragmentation (7). Apoptosis is an important defense against cancer that involves the elimination of potentially harmful cells. Numerous diseases have been associated with dysregulated apoptotic processes that ultimately lead to the inhibition of cell death and propagation of diseases, such as cancer (8).

A previous epidemiological study showed that chronic inflammation predisposes individuals to certain types of cancer (9). Hallmarks of inflammation-related cancers include the presence of inflammatory cells and mediators in tumor tissues, tissue remodeling and angiogenesis, similar to that observed during chronic inflammatory responses and tissue repair. The study of mechanisms underlying inflammation-related cancer has been focused on the early stages of cancer (10).

Previously, Kudingcha was shown to demonstrate strong *in vitro* anti-cancer effects in human nasopharyngeal carcinoma cells (11). In the present study, the anti-cancer and anti-metastatic effects of Kudingcha were further examined. MCF-7 human breast adenocarcinoma cells were treated with Kudingcha and the molecular mechanisms underlying the consequent anticancer effects were studied. Changes in the activities of Kudingcha were evaluated at different concentrations and the anti-metastatic effects were assessed in mice with tumors propagated by 26-M3.1 colon carcinoma cells.

## Materials and methods

### Preparation of Ilex kudingcha C. J. Tseng (*Kudingcha*)

Kudingcha was purchased in Chongqing, China. The Kudingcha was stored at −80°C and freeze-dried to produce a powder. A 20-fold volume of boiling water was added to the powdered sample and extracted twice. The water extract was evaporated using a rotary evaporator (N-1100; Eywla, Tokyo, Japan), concentrated and then dissolved in dimethylsulfoxide (DMSO; Amresco, Solon, OH, USA) to adjust to the stock concentration (20%, w/v).

### Cancer cell preparation

MCF-7 human breast adenocarcinoma cells obtained from the American Type Culture Collection (ATCC; Manassas, VA, USA) were used for the experiments. The cells were cultured in RPMI-1640 medium (Gibco Co., Birmingham, MI, USA) supplemented with 10% fetal bovine serum (FBS; Gibco Co.) and 1% penicillin-streptomycin (Gibco Co.) at 37°C in a humidified atmosphere containing 5% CO_2_ (model, 311 S/N29035; Forma, Waltham, MA, USA). The medium was changed two or three times each week.

### 3-(4,5-dimethyl-2-thiazolyl)-2,5-diphenyltetrazoliumbromide (MTT) assay

The anticancer effects of Kudingcha were assessed by MTT assay. The MCF-7 human breast adenocarcinoma cells were seeded in a 96-well plate at a density of 2×10^4^cells/ml in a volume of 180 *μ*l per well. Kudingcha solutions (20 *μ*l) with concentrations of 50, 100 and 200 *μ*g/ml were added and then the cells were incubated at 37°C in 5% CO_2_ for 48 h. An MTT solution (200 *μ*l, 5 mg/ml; Amresco) was added and the cells were cultured for a further 4 h under the same conditions. Subsequent to removing the supernatant, 150 *μ*l of DMSO was added per well and mixed for 30 min. Finally, the absorbance of each well was measured with an ELISA reader (model 680; Bio-Rad, Hercules, CA, USA) at 540 nm (12).

### RT-PCR to measure mRNA expression

Total RNA was isolated from the MCF-7 human breast adenocarcinoma cells using TRIzol reagent (Invitrogen, Carlsbad, CA, USA), according to the manufacturer’s instructions. The RNA was digested with RNase-free DNase (Roche, Basel, Switzerland) for 15 min at 37°C and purified using an RNeasy kit (Qiagen, Hilden, Germany), according to the manufacturer’s instructions. cDNA was synthesized from 2 *μ*g total RNA by incubation at 37°C for l h with avian myeloblastosis virus (AMV) reverse transcriptase (GE Healthcare, Little Chalfont, Buckinghamshire, UK) with random hexanucleotides, according to the manufacturer’s instructions. The sequences of the primers that were used to specifically amplify the genes of interest are shown in Table I. Amplification was performed in a thermal cycler (Eppendorf, Hamburg, Germany). The PCR products were separated in 1.0% agarose gels and visualized using ethidium bromide (EtBr) staining (13).

### Measurement of lung metastasis following Kudingcha treatment in BALB/c mice bearing 26-M3.1 colon carcinoma cell tumors

A quantity of 26-M3.1 colon carcinoma cells were obtained from Professor Yoon at the Department of Food and Nutrition, Yuhan University, Bucheon, South Korea. These highly metastatic cells were maintained as monolayers in Eagle’s minimal essential medium (EMEM; Gibco Co.) supplemented with 7.5% FBS, a vitamin solution, sodium pyruvate, non-essential amino acids and L-glutamine (Gibco Co.). The cultures were maintained in a humidified atmosphere of 5% CO_2_ at 37°C. Experimental lung metastasis was induced by injecting the colon 26-M3.1 cells into the lateral tail vein of 6-week-old female Balb/c mice (Experimental Animal Center of Chongqing Medical University, Chongqing, China) (14). Kudingcha solutions (400, 800 and 1,600 mg/kg) were subcutaneously injected into the mice and after 2 days the animals were intravenously inoculated with the 26-M-3.1 cells (2.5×10^4^/mouse). After 2 weeks the mice were sacrificed and their lungs were fixed in Bouin’s solution (saturated picric acid:formalin:acetic acid, 15:5:1; v/v/v). The rate of metastasis was assessed by counting the lung tumor colonies (tumors on the lung surface) as observed under the naked eye using a digital camera (Canon D550, Tokyo, Japan). The inhibitory rate of metastasis was assessed using the formula: Inhibitory rate = (Number of control mouse metastatic tumors − number of kudingcha mouse metastatic tumors) / number of control mouse metastatic tumors × 100. The protocol for these experiments was approved by the Animal Ethics Committee of Chongqing Medical University.

### Statistical analysis

Data are presented as the mean ± SD. Differences between the mean values for individual groups were assessed using a one-way ANOVA with Duncan’s multiple range test. P<0.05 was considered to indicate a statistically significant difference. SAS version 9.1 (SAS Institute Inc., Cary, NC, USA) was used for statistical analysis.

## Results

### In vitro anticancer effect of Kudingcha on MCF-7 cells

The anti-cancer effects of Kudingcha on the MCF-7 cells were evaluated using an MTT assay. The growth inhibitory rates of the MCF-7 cells treated with the varying concentrations of Kudingcha are shown in Table II. When solutions of the Kudingcha were administered to the MCF-7 cells, the growth inhibitory rates observed at concentrations of 50, 100 and 200 *μ*g/ml were 19, 58 and 81%, respectively (P<0.05). These results demonstrated that Kudingcha had marked anti-proliferative effects on the MCF-7 cells. In addition, it was observed that the higher the concentration of Kudingcha, the stronger the anticancer effects.

### Induction of apoptosis by Kudingcha

**Figure 1:**
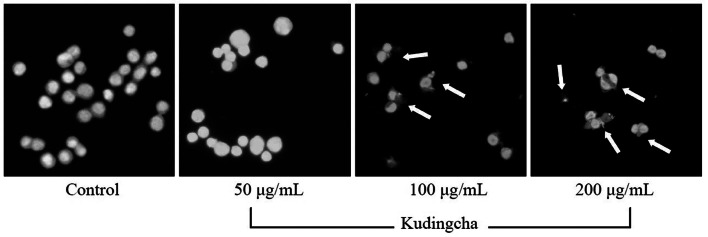
Exposure of MCF-7 human breast adenocarcinoma cells to Kudingcha induces apoptosis. Control, cancer cells were not treated with Kudingcha; Kudingcha, cancer cells were treated with Kudingcha (50, 100 and 200 *μ*g/ml); Arrows, apoptotic cells.

In order to determine a possible mechanism underlying the growth inhibitory activity of Kudingcha in the MCF-7 cancer cells, the induction of apoptosis was monitored. The extent of chromatin condensation was analyzed by fluorescence microscopy of cells stained with the DNA-binding fluorescent dye, 4,6-diamidino-2-phenylindole (DAPI). While the untreated MCF-7 cells presented nuclei with homogeneous chromatin distribution, treatment with Kudingcha induced chromatin condensation and nuclear fragmentation, suggesting the presence of apoptotic cells (Fig. 1).

### Apoptosis-related gene expression of Bax, Bcl-2 and caspases

**Figure 2:**
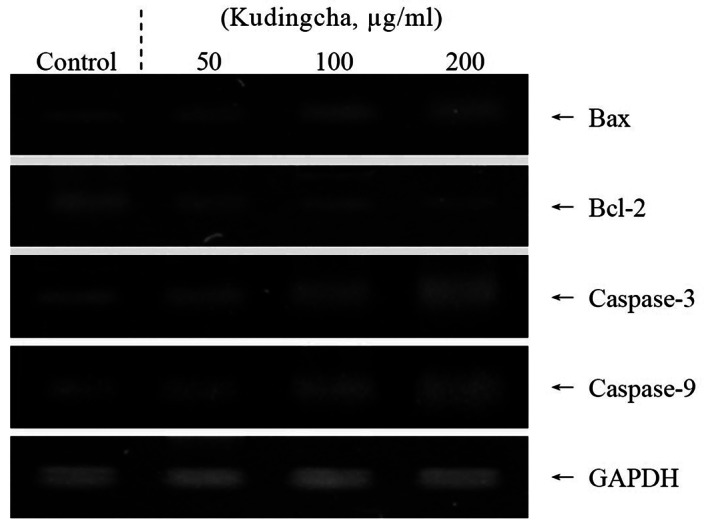
Effects of Kudingcha on the mRNA expression of Bax, Bcl-2 and caspase-3 and -9 in MCF-7 human breast adenocarcinoma cells. the expression of pro-apoptotic Bax and anti-apoptotic Bcl-2 showed significant changes in the presence of 200 *μ*g/ml Kudingcha. These results suggest that Kudingcha induced apoptosis in the MCF-7 cells via a Bax- and Bcl-2-dependent pathway.

To elucidate the mechanisms underlying the inhibition of cancer cell growth by Kudingcha, the expression of Bax, Bcl-2, and caspase-3 and -9 was measured in the MCF-7 cells by RT-PCR analysis following a 48-h incubation with the various concentrations of Kudingcha solution. As shown in Fig. 2, the expression of pro-apoptotic Bax and anti-apoptotic Bcl-2 showed significant changes in the presence of 200 *μ*g/ml Kudingcha. These results suggest that Kudingcha induced apoptosis in the MCF-7 cells via a Bax- and Bcl-2-dependent pathway. The mRNA expressions levels of caspase-3 and -9 were extremely low in the untreated control MCF-7 cells, but significantly increased once the cells were treated with 200 *μ*g/ml Kudingcha. With increased concentrations of the Kudingcha treatment, the mRNA expression of caspase-9 and -3 was gradually elevated. More specifically, the apoptotic induction caused by Kudingcha was correlated with the upregulation of Bax, caspase-3 and caspase-9, and the down-regulation of Bcl-2 in terms of mRNA expression. The effects of 200 *μ*g/ml Kudingcha were greater than those of the 100 and 50 *μ*g/ml Kudingcha solutions.

### Inflammation-related gene expression of NF-κB, IκB-α, iNOS and COX-2

**Figure 3:**
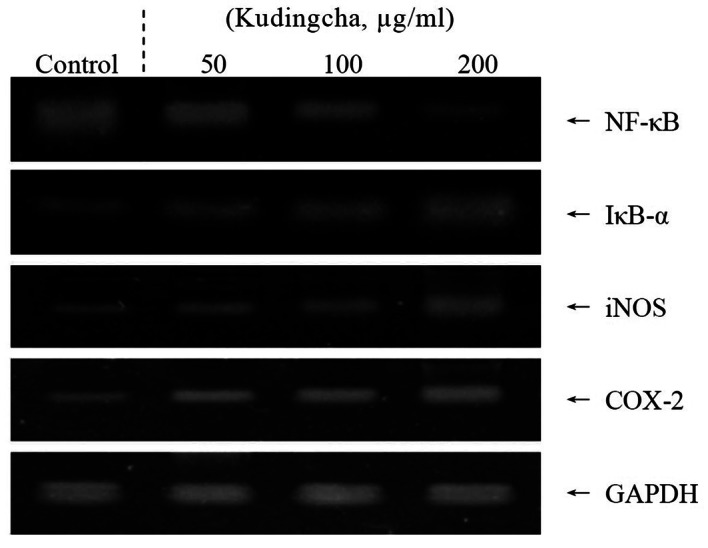
Effects of Kudingcha on the mRNA expression of NF-κB, IκB-α, iNOS and COX-2 in MCF-7 human breast adenocarcinoma cells. mRNA expression of NF-κB and IκB-α was reduced in the MCF-7 cells treated with 200 *μ*g/ml Kudingcha solution. Kudingcha significantly modulated the expression of the genes associated with inflammation.

The present study also determined whether the anticancer actions of Kudingcha were associated with the inhibition of NF-κB, IκB-α, iNOS and COX-2 gene expression. As shown in Fig. 3, the mRNA expression of NF-κB and IκB-α was reduced in the MCF-7 cells treated with 200 *μ*g/ml Kudingcha solution. Kudingcha significantly modulated the expression of the genes associated with inflammation. The mRNA expression of NF-κB was decreased, while IκB-α mRNA expression levels were increased. Additionally, the mRNA expression of COX-2 and iNOS was gradually decreased in the presence of Kudingcha depending on the concentrations. These observations indicate that Kudingcha may help prevent cancer in the early stages by increasing anti-inflammatory activities. Overall, the results of this experiment demonstrated that a higher concentration of Kudingcha had a stronger anti-inflammatory effect on the human breast adenocarcinoma cells than lower concentration solutions.

### In vivo anti-metastatic effect of Kudingcha

The prophylactic inhibition of tumor metastasis by Kudingcha was evaluated using an experimental mouse metastasis model (Table III). All Kudingcha-treated mice had significantly fewer lung metastatic colonies than those of the control mice (number of metastatic tumors, 57±6, n=10; P<0.05). Kudingcha was most effective at inhibiting lung metastasis at a concentration of 1600 mg/kg. This concentration (inhibitory rate, 33.3%; number of metastatic tumors, 38±6) inhibited tumor formation and lung metastasis to a greater degree than the 800 mg/kg (inhibitory rate, 22.8%; number of metastatic tumors, 44±6) and 4000 mg/kg solutions (inhibitory rate, 8.8%; number of metastatic tumors, 52±6).

## Discussion

Apoptosis is a fundamental cellular event, and understanding its mechanisms of action will aid in harnessing this process for use in tumor diagnosis and therapy (15). In a healthy cell, the anti-apoptotic protein Bcl-2 is expressed on the outer mitochondrial membrane surface (16). Apoptosis results from the activation of caspase family members that act as aspartate-specific proteases (17). Cytochrome *c* and procaspase-9 processing is highly dependent on caspase-3, thus, this caspase is in a central position as a regulator of the essential apoptotic pathways in cancer cells (18). Caspase-3 has also been reported to play a role as an amplifier of apoptotic signals (i.e., by cleaving Bcl-2) (19).

Additionally, anticancer mechanisms underlying the effect of Kudingcha on human cancer cells involve the induction of apoptosis by increasing the number of apoptotic bodies, regulating the mRNA expression of Bax and Bcl-2 and promoting anti-inflammatory effects by downregulating iNOS and COX-2 gene expression. COX-2 has been suggested to play an significant role in colon carcinogenesis, and NOS, along with iNOS, may be a good target for the chemoprevention of colon cancer (20). NF-κB is one of the most ubiquitous transcription factors that regulates the expression of genes required for cellular proliferation, inflammatory responses and cell adhesion (21). These mechanisms may be involved in the anticancer effects of Kudingcha in cancer cells. Based on the results of the MTT assay and the expression patterns of pro-apoptotic genes observed in the present study, we concluded that cancer cells treated with Kudingcha underwent apoptosis. With similar results to these findings, the anticancer effects of Kudingcha in human nasopharyngeal carcinoma cells were evaluated in a previous study using MTT assay and RT-PCR analysis (11).

Metastasis is defined as the spread of cancer cells from one organ or area to another adjacent organ or location (22). Malignant tumor cells are considered to have the capacity to metastasize. Cancer occurs once cells in a tissue are genetically damaged in a progressive manner, resulting in cancer stem cells possessing a malignant phenotype. Once the tumor cells come to rest in another site, they penetrate the vessel walls, continue to multiply and eventually form another tumor. Colon 26-M3.1 carcinoma cells have been used to evaluate anti-metastasis effects *in vivo*(23).

In conclusion, the present study used various *in vitro* experimental methods, including MTT assays, DAPI staining and RT-PCR assays, to evaluate the anticancer effects of Kudingcha. A mouse model bearing tumors produced by 26-M3.1 colon carcinoma cells was also assessed to study the *in vivo* effects of Kudingcha. Overall, Kudingcha demonstrated potent *in vitro* and *in vivo* anti-cancer activities, particularly for combating *in vivo* tumor metastasis. The functional contents of Kudingcha are important for augmenting these anticancer effects. A high concentration solution of Kudingcha increased the anticancer properties observed in the present study. However, the active compounds of Kudingcha require identification and evaluation in future studies.

## Figures and Tables

**Table I:** Sequences of RT-PCR primers used in the present study.

Gene name	Sequence
Bax	Forward, 5′-AAG CTG AGC GAG TGT CTC CGG CG-3′
	Reverse, 5′-CAG ATG CCG GTT CAG GTA CTC AGT C-3′
Bcl-2	Forward, 5′-CTC GTC GCT ACC GTC GTG ACT TGG-3′
	Reverse, 5′-CAG ATG CCG GTT CAG GTA CTC AGT C-3′
Caspase-3	Forward, 5′-CAA ACT TTT TCA GAG GGG ATC G-3′
	Reverse, 5′-GCA TAC TGT TTC AGC ATG GCA-3′
Caspase-9	Forward, 5′-GGC CCT TCC TCG CTT CAT CTC-3′
	Reverse, 5′-GGT CCT TGG GCC TTC CTG GTA T-3′
NF-κB	Forward, 5′-CAC TTA TGG ACA ACT ATG AGG TCT CTG G-3′
	Reverse, 5′-CTG TCT TGT GGA CAA CGC AGT GGA ATT TTA GG-3′
IκB-α	Forward, 5′-GCT GAA GAA GGA GCG GCT ACT-3′
	Reverse, 5′-TCG TAC TCC TCG TCT TTC ATG GA-3′
iNOS	Forward, 5′-AGA GAG ATC GGG TTC ACA-3′
	Reverse, 5′-CAC AGA ACT GAG GGT ACA-3′
COX-2	Forward, 5′-TTA AAA TGA GAT TGT CCG AA-3′
	Reverse, 5′-AGA TCA CCT CTG CCT GAG TA-3′
GAPDH	Forward, 5′-CGG AGT CAA CGG ATT TGG TC-3′
	Reverse, 5′-AGC CTT CTC CAT GGT CGT GA-3′

**Table II:** Growth inhibition of MCF-7 human breast adenocarcinoma cells caused by varying concentrations of Kudingcha, as evaluated by MTT assay at OD_540._

	Concentration of sample, *μ*g/ml		
Treatment	50	100	200
Control (untreated)	-	0.531±0.005^a^	-
Kudingcha	0.430±0.007^b^ (19)	0.223±0.009^c^ (58)	0.101±0.008^d^ (81)

Values in parentheses are the inhibition rates (%). Mean ± SD values with different letters in the same column are significantly different (P<0.05) according to Duncan’s multiple range test.

a P<0.05 vs. control group;

b P<0.05 vs. Kudingcha (50 *μ*g/ml) group;

c P<0.05 vs. Kudingcha (100 *μ*g/ml);

d P<0.05 vs. Kudingcha (200 *μ*g/ml) group. MTT, 3-(4,5-dimethylthiazol-2-yl)-2,5-diphenyltetrazolium bromide; OD, optical density.

**Table III:** Inhibitory effects of Kudingcha on the metastasis of tumors produced by colon 26-M3.1 cells in Balb/c mice.

Group	Number of metastatic tumors	Inhibitory rate (%)
Control	57±6^a,b^	-
Kudingcha		
400 mg/kg	52±6^c^	8.8
800 mg/kg	44±6^d^	22.8
1,600 mg/kg	38±6^e^	33.3

Mean ± SD values with different letters in the same column are significantly different (P<0.05) according to Duncan’s multiple range test.

a P<0.05 vs. control group;

b P<0.05 vs. Kudingcha (50 *μ*g/ml) group;

c P<0.05 vs. Kudingcha (100 *μ*g/ml);

d P<0.05 vs. Kudingcha (200 *μ*g/ml) group.
